# Phase-contrast x-ray tomography of neuronal tissue at laboratory sources with submicron resolution

**DOI:** 10.1117/1.JMI.7.1.013502

**Published:** 2020-02-20

**Authors:** Marina Eckermann, Mareike Töpperwien, Anna-Lena Robisch, Franziska van der Meer, Christine Stadelmann, Tim Salditt

**Affiliations:** a University of Göttingen, Institute for X-Ray Physics, Göttingen, Germany; b University of Göttingen, Cluster of Excellence “Multiscale Bioimaging: From Molecular Machines to Networks of Excitable Cells”, Göttingen, Germany; c University Medical Center Göttingen, Institute for Neuropathology, Göttingen, Germany

**Keywords:** phase-contrast tomography, nanofocus x-ray source, cone-beam geometry, neuronal tissue, human cerebellum

## Abstract

**Purpose:** Recently, progress has been achieved in implementing phase-contrast tomography of soft biological tissues at laboratory sources. This opens up opportunities for three-dimensional (3-D) histology based on x-ray computed tomography (μ- and nanoCT) in the direct vicinity of hospitals and biomedical research institutions. Combining advanced x-ray generation and detection techniques with phase reconstruction algorithms, 3-D histology can be obtained even of unstained tissue of the central nervous system, as shown, for example, for biopsies and autopsies of human cerebellum. Depending on the setup, i.e., source, detector, and geometric parameters, laboratory-based tomography can be implemented at very different sizes and length scales.

We investigate the extent to which 3-D histology of neuronal tissue can exploit the cone-beam geometry at high magnification M using a nanofocus transmission x-ray tube (nanotube) with a 300 nm minimal spot size (Excillum), combined with a single-photon counting camera. Tightly approaching the source spot with the biopsy punch, we achieve high $M\approx 10^{1}-10^{2}$, high flux density, and exploit the superior efficiency of this detector technology.

**Approach:** Different nanotube configurations such as spot size and flux, M, as well as exposure time, Fresnel number, and coherence are varied and selected in view of resolution, field of view, and/or phase-contrast requirements.

**Results:** The data show that the information content for the cytoarchitecture is enhanced by the phase effect. Comparison of results to those obtained at a microfocus rotating-anode x-ray tomography setup with a high-resolution detector, i.e., in low-M geometry, reveals similar to slightly superior data quality for the nanotube setup. In addition to its compactness, reduced power consumption by a factor of $10^{3}$, and shorter scan duration, the particular advantage of the nanotube setup also lies in its suitability for pixel detector technology, enabling an increased range of opportunities for applications in laboratory phase-contrast x-ray tomography.

**Conclusions:** The phase retrieval scheme utilized mixes amplitude and phase contrast, with results being robust with respect to reconstruction parameters. Structural information content is comparable to slightly superior to previous results achieved with a microfocus rotating-anode setup but can be obtained in shorter scan time. Beyond advantages as compactness, lowered power consumption, and flexibility, the nanotube setup’s scalability in view of the progress in pixel detector technology is particularly beneficial. Further progress is thus likely to bring 3-D virtual histology to the performance in scan time and throughput required for clinical practice in neuropathology.

## 1 Introduction

Phase-contrast x-ray tomography offers a unique potential to realize 3-D virtual histology, with cellular and even subcellular resolution, and for 3-D volumes, which are inaccessible by more established techniques. While volume and throughput in conventional histology are limited by slicing and staining, volume penetration for both light- and electron-based microscopy techniques is unsuitable for larger tissue volumes. Several propagation-based x-ray phase-contrast tomography studies with synchrotron radiation have demonstrated this potential, both for stained^1^^–^^3^ and unstained ^4^^,^^5^ soft tissues, and including mouse models^6^ as well as human tissue.^5^^,^^7^ Many different types of tissues have been imaged, including heart and cardiovascular systems,^8^^–^^10^ skin,^11^ cancerous tissue in particular for mamma,^12^ the peripheral nervous system,^1^^,^^13^ and tissues of the central nervous system.^7^^,^^14^^–^^16^

Toward broader accessibility and use of the technique in the clinical setting, translation from synchrotron to laboratory sources is an important goal of ongoing technique and instrumentation development. Laboratory-based phase-contrast computed tomography (μCT) has already been implemented at different sources: transmission microfocus sealed tubes,^17^ liquid metal-jet anodes,^16^^,^^18^^–^^21^ as well as microfocus rotating-anode sources.^22^^,^^23^ The detection technology has been equally diverse. Nanotubes offer yet another opportunity to implement phase-contrast tomography with possible advantages, in particular, in view of increased spatial coherence length. For stained tissue, high-resolution tomography of different tissues has been demonstrated in Refs. 24 and 25. A particular challenge is to reach sufficient image quality for unstained soft tissues with laboratory radiation. To this end, we have recently demonstrated the capabilities of optimized image acquisition (by geometry and detection) and reconstruction, using liquid-anode metal-jet sources as well as microfocus rotating-anode sources.^23^^,^^26^

**Fig. 1:**
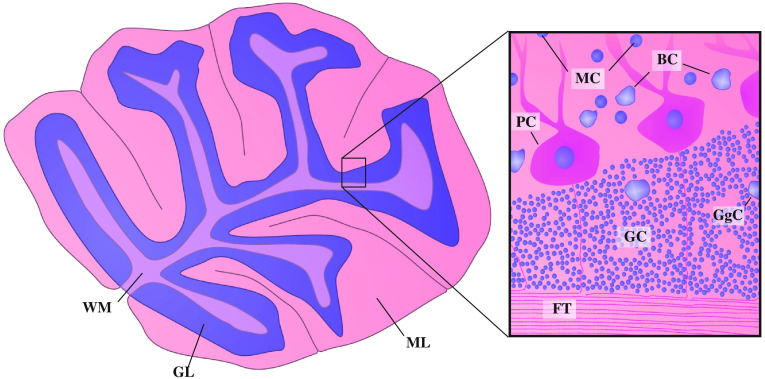
Depiction of the cerebellum anatomy, giving a rather macroscopic perspective on the left and a zoom-in to the cellular scale on the right. Notations correspond to the ones defined in the text.

In this work, we want to investigate the suitability of a home-built setup installed at a nanotube (NanoTube N1 60 kV, Excillum AB, Stockholm, Sweden) for 3-D histology of unstained “postmortem” human brain tissue. As in our earlier study,^5^ we chose cerebellar tissue as a reference structure for phase-contrast tomography data evaluation, due to its well-known anatomy and features covering various relevant length scales and $e^{-}$ densities. Figure 1 illustrates its basic anatomical features in order to place the tomographic results obtained on biopsy punches from paraffin-embedded tissue blocks with typical diameters of a millimeter into proper anatomical perspective. The “white matter” (WM) consists of myelinated neuronal structures as “axons” in “fiber tracts” (FT). From there, fibers traverse the “granular layer” (GL), consisting of “granular cells” (GC) and “Golgi cells” (GgC). Bounding the GL, a single layer of “Purkinje cells” (PC, PCL) with sparse occurrences of “basket cells” (BC) is situated. PC’s long, planar, and widely branched dendritic trees reach into the “molecular layer” (ML), which distinguishes itself from the GL by the “molecular cells” (MC), having more sparsely spread neurons and having larger nuclei.

The paper is organized as follows. After this introduction, the setup realization and data processing of the nanotube x-ray source are outlined in Sec. 2.1, complemented by a brief presentation of a well-established rotating-anode microfocus setup in Sec. 2.2. The results (Sec. 3) start with the inspection of commissioning test measurements regarding stability and spectrum in Sec. 3.1. However, the analysis of the tomographic data occupies the main part of this work, being devoted to the variation of physical parameters as source spot size, lateral coherence length, dose, or Fresnel number in Secs. 3.2.1 and 3.2.2 and also to the comparison with a well-established microfocus setup in Sec. 3.2.3. This paper closes with a summary and outlook in Sec. 4.

## 2 Methods

### 2.1 NanoTube Implementation

#### 2.1.1 Experimental setup

**Fig. 2:**
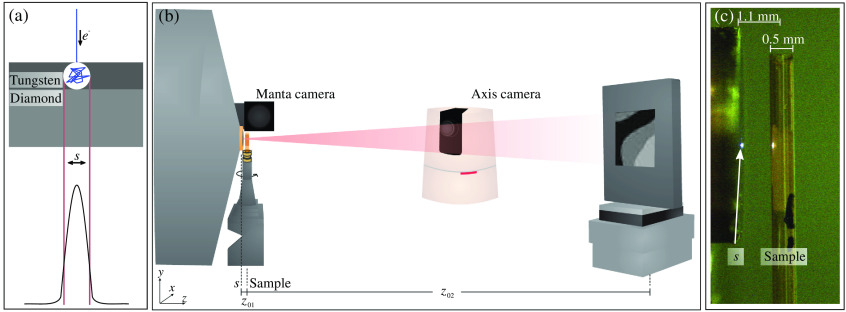
Illustration of the nanotube setup: (a) schematic of the transmission target x-ray source; (b) recording geometry: the cone-shaped front end of the nanotube allows for small distances $z_{01}$ between source s and the sample. The detector is aligned at $z_{02}$ from the source. The axis camera allows for visual inspection of the setup in general, while the Manta camera gives a microscopic control of the tight $z_{01}$ environment; see exemplary view in (c).

We have designed a nanofocus x-ray setup for propagation-based x-ray phase-contrast tomography, as depicted in Fig. 2. Its central component is the NanoTube N1 60 kV (Excillum AB) x-ray source, with two-dimensional (2-D) spatial resolution down to 150 nm according to the manufacturer (lines-and-spaces, metal test objects in absorption contrast). The source is operated at 60 keV with a power between 0.2 and 1.2 W, depending on the spot size. The NanoTube system was calibrated for three different spot sizes s [full width at half maximum, (FWHM)]: “big spot, high flux” at ∼1  μm, “middle spot, middle flux” at ∼0.5  μm, and “small spot, low flux” at ∼0.3  μm (FWHM). Figure 2(a) shows the schematic of the transmission-anode target, which consists of a 0.50  μm W-film providing a rather high $e^{-}$ stopping power S (S=54.4  MeV/cm^27^) on a 100-μm layer of diamond, serving as carrier layer and for heat mitigation at fewer $e^{-}$ interactions (S=18.1  MeV/cm). As shown in the zoomed-in optical micrograph in Fig. 2(c), samples can be positioned in direct proximity to the target, since it also serves as a vacuum exit window. The cone-shaped front end of the NanoTube allows to position the sample tower underneath the x-ray target and to achieve small source-to-sample distances down to $z_{01}\geq 100\;\mu m$. Owing to the small submicron spot size s, high geometrical magnification $M=z_{02}/z_{01}\gg 1$ can be realized without source blurring, making it possible to use direct (photon-counting) pixel detectors, which are available only with relatively large pixel size px. Accordingly, the effective pixel size in the sample plane is reduced to^28^

$$px_{\mathrm{eff}}=\frac{1}{M}\cdot px=\frac{z_{01}}{z_{02}}\cdot px.$$ (1)

At the same time, phase contrast is still feasible, since small s also assures the spatial coherence length ζ^29^ to be of the same order of magnitude as $px_{\mathrm{eff}}$

$$\zeta =\frac{\lambda \cdot z_{01}}{s}\geq px_{\mathrm{eff}},$$ (2)

where λ denotes the x-ray wavelength. The sample tower is equipped with three translational motors at the bottom (x,y,z) to position the tomographic rotation axis. The field of view (FOV) on the sample is selected by two further translational motors (x,z) on top of the rotation, enabling automated alignment routines, as described in Ref. 23. For high-resolution optical monitoring of the sample environment, a Manta camera (Allied Vision Technologies GmbH, Stadtroda, Germany) is installed with a view along the x axis, as shown in Figs. 2(b) and 2(c). Broad overviews of the scene are provided by an Axis camera (Axis Communications AB, Lund, Sweden). A single-photon counting Timepix Hexa H05-W0154 detector (XIE, Freiburg, Germany) was used with a 500-μm Si sensor, px=55  μm and 768×512  pixels (w×h) distributed over six modules to record the 2-D projections.

#### 2.1.2 Data recording settings

For tomographic acquisition, 2-D x-ray projections were recorded at 1201 rotation angles, equally distributed over 192 deg (due to the cone-beam angle of 12 deg, an increased angular sampling range was required), with sets of 25 flat-field images before and after each scan. The $z_{02}\approx 20\;\mathrm{cm}$ was kept fixed, varying $z_{01}$ and s. The detector was operated with a lower-limit cut-off energy of 4 keV. The acquisition time was adjusted depending on the spot size s to avoid overexposure, which is at maximum counts of 11810 ph. Most scans were split into four consecutive tomographic scans with 1/4 exposure time each, and then recombined via cross correlation in Fourier space.^30^ Thus, for these cases, four different exposures were recorded and averaged for each projection. All scan parameters are tabulated in Tables 1–3. This acquisition scheme was found to reduce ring artifacts arising from a statistically varying response of the modules.

**Table 1:** Parameters and quality measured for the tomographic scans at fixed F. Exposure times also indicate whether the scan was performed as a single or multiple ones. FSC is based on volumes of $400^{3}\;\text{voxels}$, with a Kaiser–Bessel window of 7 pixels.^31^ The SNR was calculated as $(\mu_{\mathrm{ft}}-\mu_{\mathrm{bg}})/\sigma_{\mathrm{bg}}$ on five GC nuclei, four PC bodies, and their nuclei, respectively. Scan time refers to the full scan, including readout and motor positioning.

Experimental parameters								
s (μm)	F	$z_{01}$ (mm)	$px_{\mathrm{eff}}$ (μm)	ζ (μm)	Exp. time (s)	Power (W)	Scan time (h)	Dose (kGy)
0.95	1.97	3.5	0.959	0.501	1·3.5=3.5	1.18	2.0	10.6
0.51	1.96	3.5	1.067	0.934	4·5=20	0.24	8.0	14.3
Quality measures								
s (μm)	F	$\alpha_{\mathrm{BAC}}$	$\gamma_{\mathrm{BAC}}$	FSC (μm)	SNR—GC nucleus	SNR—PC nucleus	SNR—PC body	
0.95	1.97	0.12	0.16	1.83	5.46	5.49	0.74	
0.51	1.96	0.10	0.16	1.76	6.39	9.25	2.74	

**Table 2:** Analysis of image quality as a function of s and F. Exposure times also indicate whether the scan was performed as a single or multiple ones. FSC evaluation is based on volumes of $K^{3}$ voxels, with a Kaiser–Bessel window of 7 pixels.^31^ SNR was calculated as $(\mu_{\mathrm{ft}}-\mu_{\mathrm{bg}})/\sigma_{\mathrm{bg}}$ on five GC nuclei, four PC bodies, and their nuclei, respectively.

Experimental parameters								
s (μm)	F	$z_{01}$ (mm)	$px_{\mathrm{eff}}$ (μm)	ζ (μm)	Exp. time (s)	Power (W)	Scan time (h)	Dose (kGy)
0.95	1.97	3.5	0.959	0.501	4·3.5=14	0.91	8.0	40.5
0.51	1.96	3.5	1.067	0.934	4·5=20	0.24	8.0	14.3
0.51	1.40	2.5	0.687	0.667	4·5=20	0.22	8.0	26.0
0.51	0.83	1.5	0.410	0.400	4·5=20	0.24	8.0	77.1
0.30	0.60	1.1	0.300	0.680	4·20=80	0.91	32.5	189.2
Image quality parameters								
s (μm)	F	$\alpha_{\mathrm{BAC}}$	$\gamma_{\mathrm{BAC}}$	FSC (μm)	SNR—GC nucleus	SNR—PC nucleus	SNR—PC body	
0.95	1.97	0.12	0.16	$1.00^{K=100}$	6.47	5.93	0.86	
0.51	1.96	0.10	0.16	$1.76^{K=400}$	6.39	9.25	2.74	
0.51	1.40	0.03	0.16	$1.13^{K=130}$	6.18	6.91	1.24	
0.51	0.83	0.015	0.16	$1.19^{K=200}$	5.57	5.52	1.38	
0.30	0.60	0.015	0.16	$1.02^{K=200}$	3.82	3.81	0.83	

**Table 3:** Analysis of the tomographic scans for nanofocus setup with $px_{\mathrm{eff}}\approx 1.07\;\mu m$, in comparison to a rotating-anode scan with the same $px_{\mathrm{eff}}$. Exposure times also indicate whether the scan was performed as a single or multiple ones. FSC analysis is based on volumes of $400^{3}\;\;\text{voxels}$, with a Kaiser–Bessel window of 7 pixels.^31^ SNR was calculated as $(\mu_{\mathrm{ft}}-\mu_{\mathrm{bg}})/\sigma_{\mathrm{bg}}$ on five GC nuclei, four PC bodies, and their nuclei, respectively.

Experimental parameters							
s (μm)	F	$z_{01}$ (mm)	$px_{\mathrm{eff}}$ (μm)	ζ (μm)	Exp. time (s)	Power (W)	Scan time (h)
0.51	1.96	3.5	1.067	0.934	4·5=20	0.24	8.0
70.0	1.24	494.0	1.070	1.094	1·50=50	1400	16.7
Image quality parameters							
s (μm)	F	$\alpha_{\mathrm{BAC}}$	$\gamma_{\mathrm{BAC}}$	FSC (μm)	SNR—GC nucleus	SNR—PC nucleus	SNR—PC body
0.51	1.96	0.10	0.16	1.76	6.39	9.25	2.74
70.0	1.24	0.07	0.16	1.82	5.36	6.80	2.55

#### 2.1.3 Phase retrieval

The data are recorded in the “direct contrast” or “edge enhancement regime” at Fresnel numbers:

$$F=\frac{px_{\mathrm{eff}}^{2}}{z_{\mathrm{eff}}\lambda }=\frac{px^{2}}{z_{12}M\lambda }\approx 1,$$ (3)

where $px_{\mathrm{eff}}=px/M$ for the effective pixel size and $z_{\mathrm{eff}}=z_{12}/M$ have been used for the effective propagation distance (Fresnel scaling theorem^28^). As demonstrated in Refs. 20 and 32, in this regime, even data from low coherence sources can be successfully reconstructed based on the “Bronnikov-aided correction” (BAC) scheme. To this end, the “transport of intensity equation” (TIE) serves as a starting point to describe the propagation of a paraxial wave along $\vec{z}$ with intensity $I(\vec{r})$ and phase distribution $\phi (\vec{r})$: $\nabla_{⊥}[I(\overset{\to }{r})\cdot \nabla_{⊥}\phi (\overset{\to }{r})]=-k\partial_{z}I(\overset{\to }{r})$, with k being the wavenumber. Under the assumption of small propagation distances $z_{\mathrm{eff}}$ and a purely phase-shifting object, the TIE can be linearized, and an approximate phase $\tilde{\phi }$ can be computed as^33^^,^^34^

$$\overset{˜}{\phi }({\overset{\to }{r}}_{⊥})=2\pi F\cdot F_{⊥}^{-1}\{\frac{F_{⊥}[\frac{I({\overset{\to }{r}}_{⊥},z)}{I_{0}}-1]}{{\left|{\overset{\to }{k}}_{⊥}\right|}^{2}+\alpha }\},$$ (4)

where F denotes the Fourier transform, $I_{0}$ denotes the (uniform) intensity distribution of the illumination, and k is the spatial frequency in units of ${px}^{-1}$. The parameter α is introduced to regularize the singularity at zero spatial frequencies, and in practice, is chosen such that edge enhancement is cancelled. In a second step, the approximate phase $\tilde{\phi }$ is used to compute a (corrected) sharp intensity distribution in the object exit plane, according to^35^

$$I({\overset{\to }{r}}_{⊥},z=0)=\frac{I({\overset{\to }{r}}_{⊥},z)}{1-\gamma \nabla_{⊥}^{2}\overset{˜}{\phi }({\overset{\to }{r}}_{⊥})}.$$ (5)

In practice, the relevance of using the BAC is to choose the two regularization parameters α and γ such that the resulting somewhat hypothetical intensity distribution contains contributions from intensity and phase contrast, even though expressed only in terms of transmitted intensity. As for reconstructions assuming a homogeneous object, the two contributions cannot be separated. For high-resolution laboratory μ- and nano-CTs, this scheme yields unparalleled image quality. Afterward, the phase-retrieved 2-D projection data are processed for ring artifact mitigation according to Ref. 36 and then recombined to a 3-D volume using the ASTRA-toolbox.^37^^,^^38^ Visualization was done with Avizo (Thermo Fisher Scientific, Waltham, Massachusetts).

### 2.2 Rotating-Anode Setup

For comparison, the samples were also scanned at a (home-built) laboratory μCT setup installed at a rotating Cu-anode x-ray source (Rigaku, Tokyo, Japan) with main line 8.048 keV ($K_{\alpha }$) and source size s=70  μm.^23^ It was operated at 40 keV and 30 mA. A high resolution, lens-coupled single crystal scintillator CCD detector (Xsight, Rigaku, Prague, Czech Republic), resulting in px=0.54  μm was used. Images were recorded at M=500  mm/494  mm≈1 to achieve a spatial coherence ζ≈1  μm reasonable for micron resolution (after 2×2  pixel-binning).

The number of projection angles was identical to that of the nanotube scans, distributed over an angular range of 180 deg from the cone-beam angle of ≈0  deg. The exposure time, however, needed to be increased significantly due to the very different detector technology, i.e., to 50 s. In addition to the flat fields, 10 dark images were recorded prior to each scan to account for the dark current of the CCD. With F=1.24, BAC also applies here.

## 3 Results

### 3.1 Commissioning Tests

**Fig. 3:**
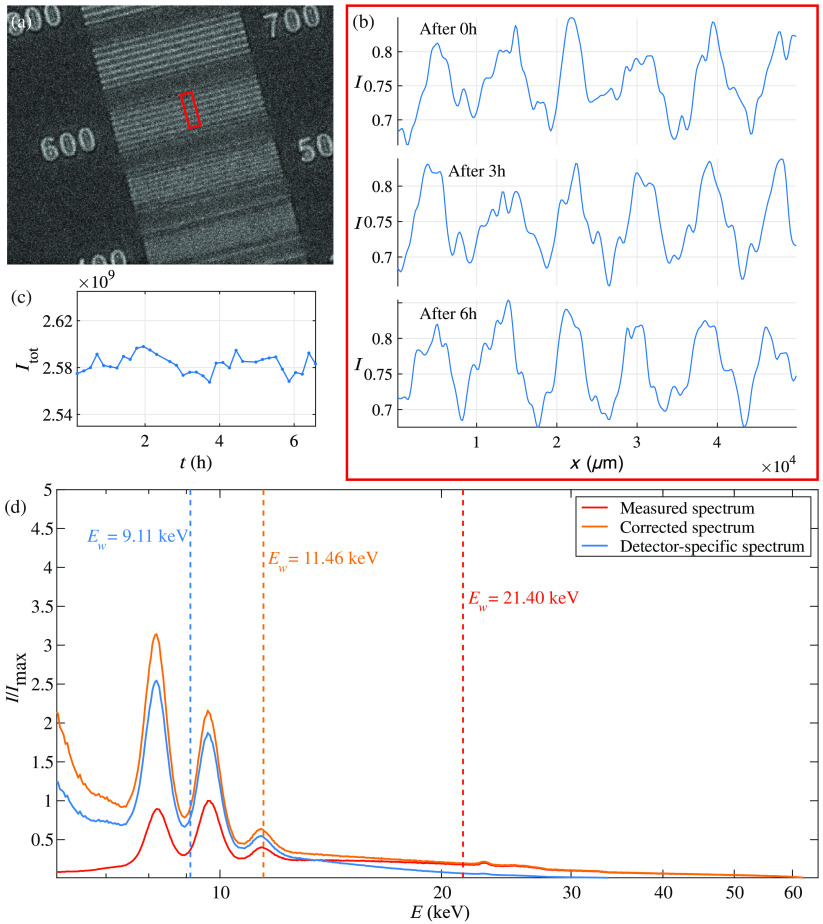
Pre-characterization of the imaging setup. (a)–(c) Test of stability by recording a time series for a test pattern, recorded at s=0.56  μm and $px_{\mathrm{eff}}=0.099\;\mu m$. (a) Image of the JIMA target (1.5  μm W). The annotation “600” refers to the structure measured, i.e., 600 nm lines and 600 nm spaces. The red rectangle indicates the area for which line profiles are shown in (b) at three time points: (top) 0 h, (center) 3 h, and (bottom) 6 h. (c) Integrated intensity over time. (d) Source spectrum: (red) measured data (detector output), (yellow) corrected for absorption in air, and (blue) the spectrum taking into account the energy dependence in the quantum efficiency of the detector used for the tomographic scans.

Prior to performing high-resolution tomography scans, the stability of the source and its environment was verified. To this end, the source was operated at spot size s=0.56  μm, and images of a custom-fabricated JIMA target (1.5  μm W; ZonePlates Ltd, London), positioned at $z_{01}=19.6\;\mathrm{mm}$, were recorded at $z_{02}=900\;\mathrm{mm}$, using an flat panel detectors camera (Photonic Science, UK) with a 5 −μm Gadox scintillator 2208×2744  pixels (w×h) and px=4.54  μm. As indicated in Fig. 3(a), images of 600 nm lines-and-spaces were acquired every 12 min for 5 min of exposure over the course of 6.6 h. Figure 3(b) shows the modulations of the lines-and-spaces corresponding to the marked area in Fig. 3(a) after 0, 3, and 6 h (from top to bottom). Only a minor shift on the order of 30  nm/h is observed. At the same time, the variations of the total intensity (integrated flat fields) shown in Fig. 3(c) are about 0.6% (RMS value).

Next, the spectrum of the x-ray source was assessed using an energy-resolving XR-100CdTe detector (Amptek, Bedford) with a sensor thickness of 1 mm, positioned at $z_{02}=1.2\;m$ to avoid saturation. Spectral bins were calibrated based on the $K_{\alpha }$ and $K_{\beta }$ fluorescence signals of Mo, Ni, and Ag foils. The spectrum is plotted in red in Fig. 3(d). The counts-weighted mean energy of this spectrum $E_{w}$ is 21.40 keV. However, at the given distance, absorption in air is already substantial and has to be corrected for using the tabulated values (Henke tables), accessed through the CXRO data base^39^ and matched to the detector bins with a MATLAB-implementation of shape-preserving piecewise cubic extrapolation. The resulted corrected curve is shown in yellow, representing the source emission spectrum, with a correspondingly lower mean energy $E_{w}=11.46\;\mathrm{keV}$. This spectrum was further corrected for the X-ray absorption in air corresponding to the detector position $z_{02}\approx 20\;\mathrm{cm}$ and for the detector sensitivity: the sensor material was 500  μm of Si, hence exhibiting a stronger sensitivity for low-energetic photons. The final, weighted spectrum is given in blue. It is characterized by $E_{w}=9.11\;\mathrm{keV}$ and therefore, represents the mean photon energy for the tomography data (without taking beam hardening in the sample into account).

The photon flux is dependent on s and the actual, specific source calibration. For s=0.95  μm, it was found to be on the order of $∼2\cdot 10^{11}\;\mathrm{ph}/s$ in 2π space, $∼4\cdot 10^{10}\;\mathrm{ph}/s$ for s=0.51  μm, and $∼1\cdot 10^{10}\;\mathrm{ph}/s$ for s=0.30  μm.

### 3.2 Phase-Contrast Tomography of Unstained Human Brain Tissue

In this work, paraffin-embedded human cerebellum was used to investigate the suitability of the setup for 3-D virtual histology and neuropathology. From the tissue block, a 0.5-mm biopsy punch was extracted and transferred into a 0.5-mm polyimide tube (Professional Plastics, Fullerton, California) on a custom-fabricated Huber brass pin (Huber Diffraktionstechnik GmbH & Co. KG, Rimsting, Germany), as illustrated in Fig. 2.

#### 3.2.1 Variation of s/ζ at constant F

**Fig. 4:**
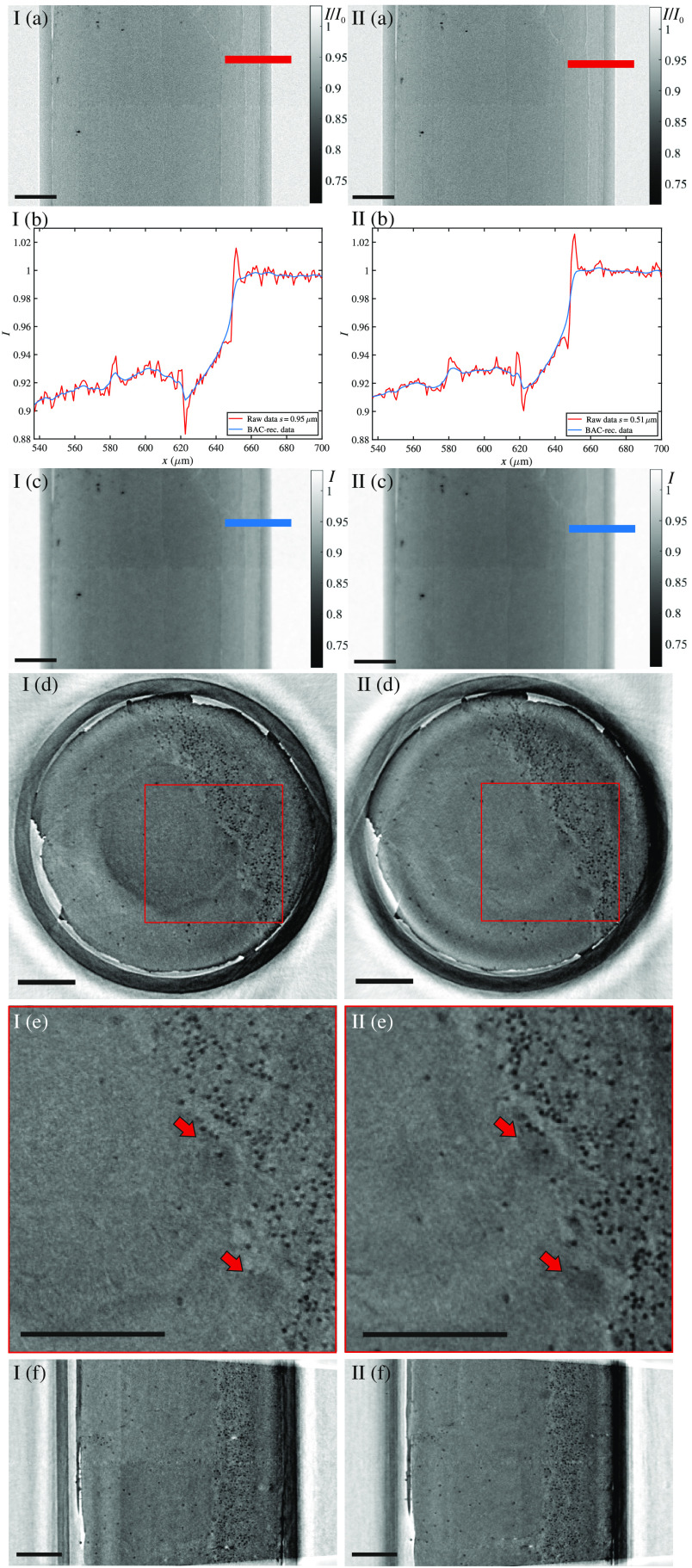
Data from tomography scans at fixed Fresnel number of 2.0, at (I) s=0.95  μm (ζ=0.501  μm) and (II) 0.51  μm (ζ=0.934  μm). (a) Respective flat-field-corrected projections and (c) BAC-reconstructed projections. Colored bars indicate the position of the profiles shown in (b). The same virtual slice (d) in the xz plane with a zoom-in in (e) and (f) in the xy plane. Red arrows mark PCs. Data from quantitative analysis are summarized in Table 1. Scale bars: 100  μm.

We have first investigated the influence of the lateral coherence length ζ on the tomographic image quality by setting two different spot sizes (I) s=0.95  μm and (II) s=0.51  μm, respectively, while keeping F≈2.0 constant, and the dose also approximately at the same level. Hence the larger source size resulted in a significantly reduced total scan time. All experimental and phase reconstruction parameters are listed in Table 1. In this work, the radiation dose was calculated combining the photon counts from the projection data with the spectra in Fig. 3. Figure 4 illustrates the data reconstruction steps from projection to orthoslices through the reconstructed volume. The projections in Fig. 4(a) reveal slight edge enhancement in both cases, especially for the polyimide–air interface, as plotted in Fig. 4(b). As expected, the higher coherence for the 0.501−μm source spot yields a more pronounced edge enhancement compared to 0.934  μm. In the same plot, the blue curves show the profiles after phase retrieval, and the respective 2-D images are given in Fig. 4(c). Virtual slices through the same position in the xz plane and xy plane are shown in Figs. 4(d) and 4(f), respectively, with a corresponding zoom in shown in Fig. 4(e). Data for (I) were recorded as a single tomographic scan, acquisition for (II) was split into four as described in Sec. 2.1. By fractionating the dose over four scans, ring artifacts were found to be reduced. Based on visual inspection, both data sets appear to be of very similar quality. Different cerebellum-specific regions, as outlined in Fig. 1, are clearly identifiable: as for the zoom ins (I.e) and (II.e), the cell-dense GL on the right and the ML on the left are separated by the sparsely distributed, bold PCL cells (red arrows). The resolution was quantified exploiting Fourier Shell Correlation (FSC).^40^^,^^41^ In this analysis scheme, two independently recorded Kaiser–Bessel-filtered data are evaluated for their consistency, defined via the intersection of the cross correlation in Fourier space with a threshold criteria of 1/2-bit in this case.^42^ Therefore, FSC values are not only governed by spatial resolution but also by the overall noise level. In this work, two independent data sets are reached performing the tomographic reconstruction twice, using half of the angular projections each. Hence, FSC-based resolution evaluation serves as an upper limit estimate. For both scans inspected here, the analysis results in similar values of around ∼1.8  μm. However, case (I) for s=0.51  μm has superior feature contrast (see Table 1).

#### 3.2.2 Variations of F at constant source spot size s

**Fig. 5:**
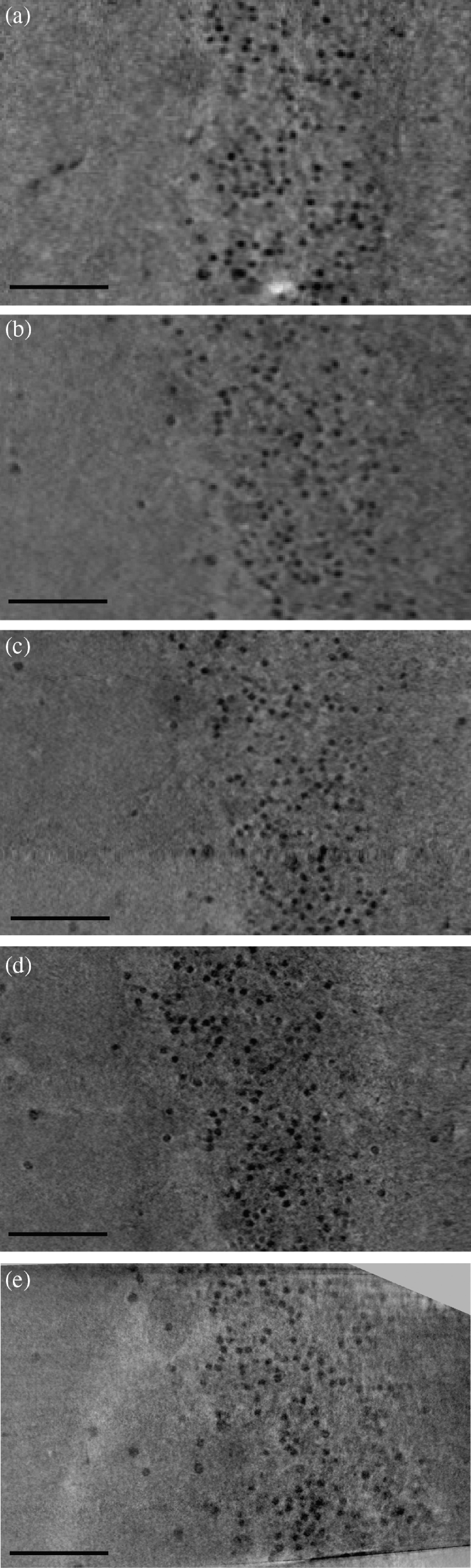
Virtual xy slices through the tomographic reconstruction at similar positions in the biopsy punch for spot sizes of (a) 0.95  μm, (b)–(d) 0.51  μm, and (e) 0.30  μm and Fresnel numbers of (a) 1.97, (b) 1.96, (c) 1.40, (d) 0.83, and (e) 0.60. Note the rise in geometrical magnification from (a) and (b) to (e). The respective analysis parameters are given in Table 2. Scale bars: 50  μm.

Next, we investigate the influence of F on tomographic image quality, as controlled by the source-to-sample distance $z_{01}$, while keeping $z_{02}$ fixed. Hence $px_{\mathrm{eff}}$ [Eq. (3)] and ζ [Eq. (2)] vary accordingly. Most scans were recorded at s=0.51  μm=const. In addition, we include a scan at the minimally achievable s=0.30  μm, as well as at s=0.95  μm. For the latter, the 0.95  μm scan from Table 1 was repeated four times to reach an equal overall scan time of ζ. All parameters are detailed in Table 2. Figure 5 shows the virtual slices along the xy plane through the reconstruction volume in similar positions. By repeating the 2  h-0.95  μm-scan from Table 1 four times, and increasing the dose accordingly, the 3-D resolution (FSC) is increased to 1.00  μm, i.e., close to the voxel size. The signal-to-noise ratio (SNR) also increased for GCs and PC nuclei, but only marginally for PC bodies. To increase the SNR of PC bodies substantially, it was necessary to double ζ (via reduction of s) at constant F (constant $z_{01}$). Despite the significantly lower dose, which compromised the resolution, the contrast for the rather large cell bodies is higher. As an overall trend, reducing F (via $z_{01}$) results in increased resolution, which is expected based on higher dose (smaller $px_{\mathrm{eff}}$). At the same time, despite the rise in dose, the SNRs are lower, indicating that the increase in ζ is more important, in particular for intrinsically low-contrasted features such as PC bodies. This conclusion is also confirmed by comparing the results in Table 2 for s=0.95  μm and s=0.30  μm. In these scans, a roughly similar coherence length ζ≈0.5 to 0.7  μm results in a constant SNR≈0.83 to 0.86 for PC bodies, even though the radiation dose deviates by more than a factor of 4.

#### 3.2.3 Comparison with data from microfocus laboratory setup

**Fig. 6:**
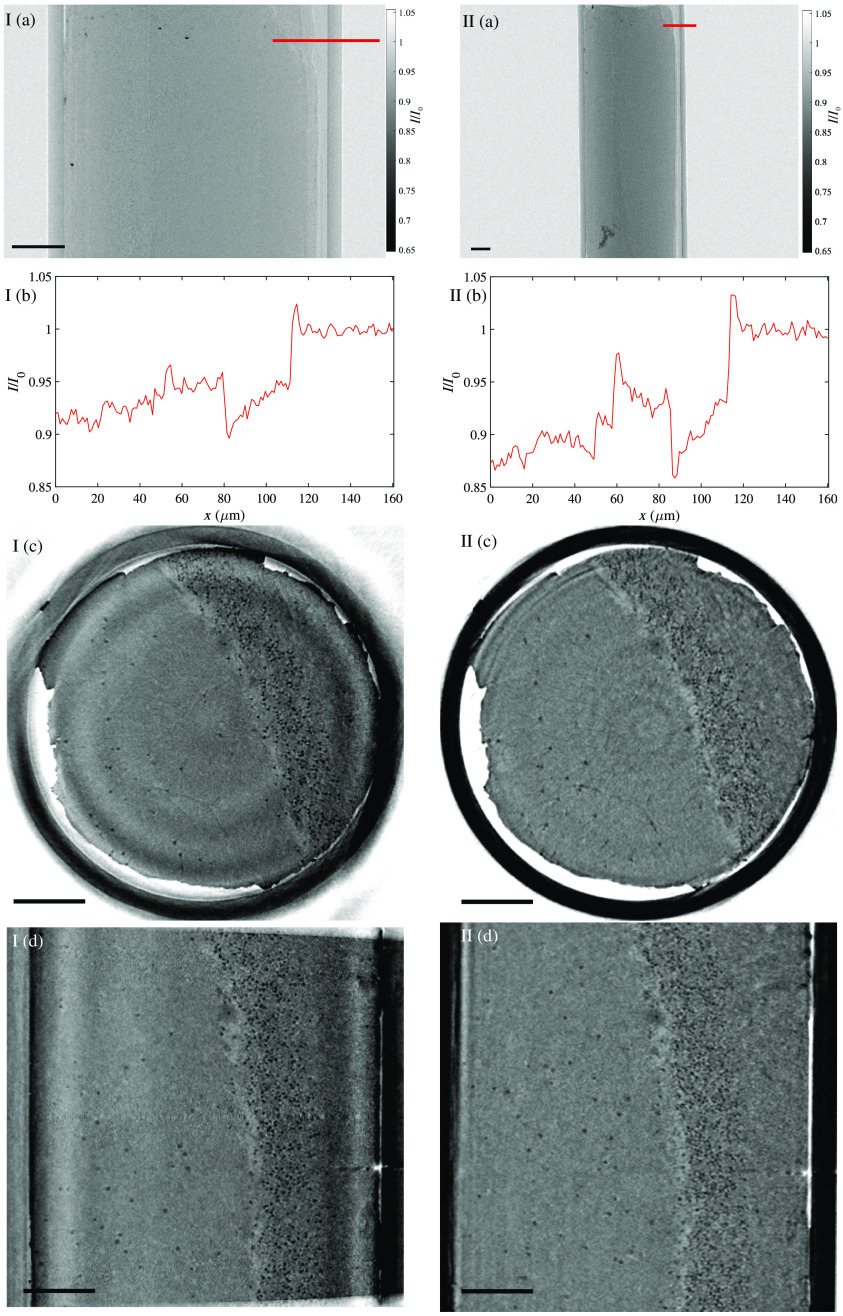
Comparison of (I) nanotube tomography with (II) microfocus rotating-anode results. Settings were chosen such that $px_{\mathrm{eff}}\approx 1.07\;\mu m$ same for both data sets. (a) Flat-field-corrected projections. The colored bars show the position of the intensity profile plots in (b). Note the identical ranges of the y axis. Virtual slices through the same position in the reconstructed sample volume are shown in (c) for the xz plane and (d) the xy plane. Scale bars: 100  μm.

To put the nanotube results into perspective with earlier implementations of 3-D histology with laboratory x-ray phase-contrast tomography, Fig. 6 and Table 3 present a comparison to a reconstruction obtained at the microfocus rotating-anode x-ray source with instrumentation described in Sec. 2.2. For the comparison, we selected the nanotube data set recorded for s=0.51  μm and F=1.96 in order to achieve similar effective pixel size $px_{\mathrm{eff}}\approx 1.07\;\mu m$ and coherence length ζ≈0.93 to 1.09  μm. The nanotube setup achieves slightly higher resolution (FSC-based) and increased SNRs (for the features considered), but requires only half of the scan time. However, as is directly apparent from Fig. 6(a), this comes at the cost of reduced FOV, reflecting the different detector technologies. For sufficiently narrow samples, this could be compensated by consecutively scanning two volumes and stacking them, which would result in similar overall scan time for both setups. The plots in Fig. 6(b) show profiles across the capillary edges indicated in Fig. 6(a). The two setups give very similar intensity profiles; absorption and edge enhancement are slightly more pronounced for the microfocus rotating-anode data. Finally, Figs. 6(c) and 6(d) show virtual slices through the xz and xy planes to judge image quality by visual inspection.

## 4 Summary and Outlook

**Fig. 7:**
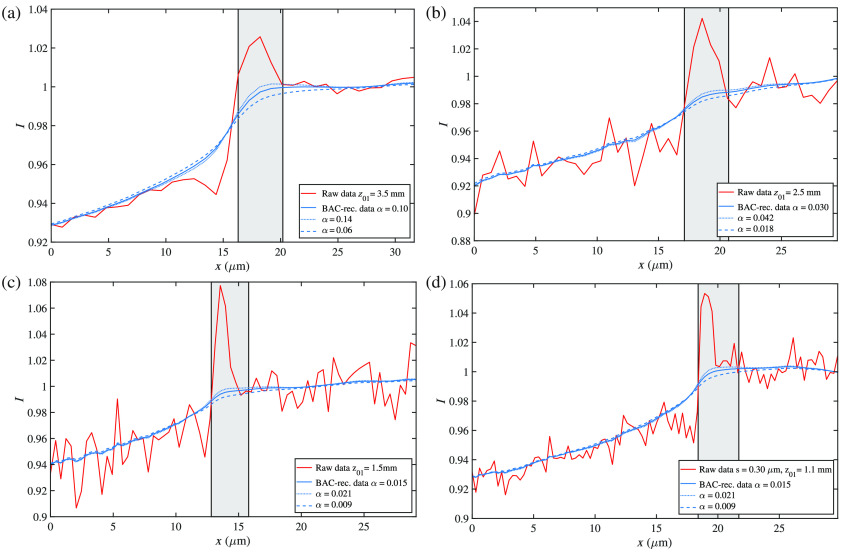
Edge enhancement and choice of reconstruction parameters. The plots correspond to the data shown in Fig. 5 and give the image profiles before reconstruction (red) exhibiting pronounced edge enhancement between air and Kapton tubes as a hallmark of phase contrast. In addition, profiles are shown after BAC reconstruction, carried out for three different values of $\alpha_{\mathrm{BAC}}$, to illustrate the process of finding the best-suited parameter value.

In summary, we have successfully demonstrated phase-contrast tomography of unstained neuronal tissue using a home-built laboratory setup with a nanofocus x-ray transmission tube (nanotube) and a photon-counting pixel detector. Sufficient image quality for the detection of neurons and hence representation of the cytoarchitecture was achieved. In particular, FSC analysis indicated a resolution of 0.90  μm for a source setting of s=0.30  μm. The phase retrieval and reconstruction scheme presented here mixes amplitude and phase information but is very suitable to visualize the small electron density differences in unstained tissue and hence the cytoarchitecture, for example, of neuronal tissue. Final results can be represented as β or δ up to a factor of k, expressing the amplitude (real-valued transmission function) or the phase shift, respectively. Importantly, the information content for the cytoarchitecture is enhanced by the phase effect, as evidenced by the variation of F (see also Fig. 7). Experimental determination of $\alpha_{\mathrm{BAC}}$, as illustrated in Fig. 7, is unproblematic and results are robust with respect to variations up to about ±40%. Since magnification and cone-beam angle are high, cone-beam tomographic reconstruction according to Ref. 43 is required. Effects of the cone-beam geometry can easily appear in the form of distortions, such as at the edges of the source-facing side of the reconstructed volume [cf. Figs. 6(I.d) and 6(II.d)]. Compared to a microfocus rotating-anode setup, data quality and information content appear to be on a similar to slightly superior level. However, the particular advantage of the nanotube setup (apart from its more compact size and much reduced power consumption) is in its scalability with respect to progress in pixel detector technology. While already performing on the same level for a relatively thin silicon sensor, future replacement by 1-mm-thick Si sensors or even GaAs sensors, along with an increase in the detection panels, may result in significantly reduced scan times. We also note that new pixel detector technology allows for registration of counting with subpixel registration and for photon energy determination,^44^ opening up an entirely new opportunity for laboratory phase-contrast tomography. Further progress is hence likely to bring virtual 3-D histology to the performance in scan time and throughput required for clinical practice in neuropathology. This, however, would be useful only in combination with automated evaluation of cytoarchitecture as in Ref. 5 and more knowledge on alterations associated with pathologies. Importantly, the demonstrated laboratory-based image quality would already be sufficient for this task.
